# Patient responsiveness as a safewards fidelity indicator: a qualitative interview study on an acute psychiatric in-patient ward

**DOI:** 10.1186/s12913-024-11326-z

**Published:** 2024-08-12

**Authors:** Veikko Pelto-Piri, Lars Kjellin, Gabriella Backman, Karoline Carlsson, Anna Björkdahl

**Affiliations:** 1 University Health Care Research Center, Faculty of Medicine and Health, Örebro University, Örebro, Sweden; 2 Psychiatric outpatient clinic, Region Värmland, Kristinehamn, Sweden; 3 Psychiatric Outpatient Clinic, Västra Götalandsregionen, Alingsås, Sweden; 4 Centre for Psychiatry Research, Department of Clinical Neuroscience, Karolinska Institutet, Stockholm, Sweden

**Keywords:** Psychiatry, Inpatients, Vulnerable populations, Coercive measures, Violence, Prevention, Implementation, Safewards.

## Abstract

**Background:**

The Safewards model aims to reduce conflict and use of containment on psychiatric wards. To evaluate the implementation of Safewards and understand why it is effective in some settings but not in others, it is important to assess the level of implementation fidelity. To do this, the Safewards Fidelity Checklist (SFC) is often used, which focuses on objective visual observations of interventions but does not include patient responsiveness. The latter is a key indicator of implementation fidelity and includes engagement, relevance, acceptability and usefulness. The aim of the present study was to investigate the fidelity of Safewards implementation on an acute psychiatric ward from the perspective of patient responsiveness.

**Method:**

The study was conducted on a ward for patients with mainly affective disorders. To assess the general level of fidelity the SFC was used together with a detailed ward walkthrough. Ten patients were interviewed with a focus on patient responsiveness to each of the seven interventions implemented on the ward. Data were analysed using qualitative descriptive analysis.

**Results:**

The findings indicate high implementation fidelity, which was reflected in the SFC assessment, walkthrough and patient responsiveness. Patients gave examples of improvements that had happened over time or of the ward being better than other wards. They felt respected, less alone, hopeful and safe. They also described supporting fellow patients and taking responsibility for the ward climate. However, some patients were unfamiliar with a ward where so much communication was expected. Several suggestions were made about improving Safewards.

**Conclusions:**

This study confirms previous research that patient responsiveness is an important factor for achieving fidelity in a prevention programme. The patients’ descriptions of the acceptability, relevance and usefulness of the specific interventions reflected to a high degree the objective visual observations made by means of the SFC and ward walkthrough. Patient engagement was demonstrated by several suggestions about how to adapt the interventions. There is potential to obtain valuable input from patients when adapting Safewards in practice. This study also presents many examples of practical work with these interventions and the effects it can have on patients’ experiences of care.

**Supplementary Information:**

The online version contains supplementary material available at 10.1186/s12913-024-11326-z.

## Background

The use of coercion in European psychiatric and mental health services has been extensively criticized for violating the UN General Assembly agreement on the Convention on the Rights of Persons with Disabilities and the Convention Against Torture and Other Cruel, Inhuman or Degrading Treatment or Punishment [1–3]. An implication of these conventions is that states should actively work towards finding ways to minimize or even abolish policies that allow coercive treatment and other coercive measures within the psychiatric services. However, in Sweden as in most Western countries, legislation still enables the use of coercive measures when certain criteria are deemed to be present [4]. Although often controversial, the aim of these pieces of legislation is to prevent harm, initiate necessary treatment and allow the patient to recover and accept continuous care on a voluntary basis.

Patients have often experienced psychiatric wards as unsafe places with shortcomings in therapeutic relationships and strict but unpredictable rules [5–7]. Patients have also reported being exposed to harm and re-traumatization during care episodes, which jeopardizes the recovery process [6, 8–10]. For staff, working in an environment that includes violence and coercive measures can cause emotional and psychological problems as well as an increased risk of long-term sick leave [11, 12]. There is a relationship between the use of coercive measures and violence in psychiatric services that has been suggested to create a negative spiral of risk and incident escalation in which patient aggression may lead to restrictive practices, which in turn may trigger further patient aggression [13]. However, the incidence of violence and coercive measures can be prevented by, for example, the use of therapeutic relationship strategies and improving the ward culture [14].

Historically, Sweden has employed more coercive measures in psychiatric care compared to many other European countries [15]. The Swedish government has faced criticism from the Committee of the Convention on the Rights of Persons with Disabilities for its excessive use of coercive measures [16]. During recent decades, several nationwide projects aimed at reducing violence and restrictive practices in inpatient psychiatry have been initiated and funded by the Swedish government. Nevertheless, according to national registers, coercive measures such as the use of seclusion, restraint and involuntary medication have not been reduced [17]. Internationally, various promising alternative interventions have been developed for psychiatric in-patient services. Most countries do not have any mandatory legislation or policies to ensure that these interventions are implemented, and they are rarely evaluated on a large scale [18, 19]. Currently, one of the best known hospital-based programmes is the Safewards model [13, 18]. Safewards, which includes ten interventions, focuses on preventing conflict and the use of containment (Table 1) [5, 20, 21]. However, implementation is complex, as in order to change the service culture, the model comprises several parallel interventions involving both staff and patients [14, 18, 19].

**Table 1:** The ten safewards interventions for reducing conflict, Fletcher et al. p. 3 [5]

Intervention	Description	Purpose
Discharge Messages	Before discharge, patients leave messages of hope for other patients on a display in the unit.	Strengthens patient community, generates hope.
Know Each Other	Patients and staff share some personal interests and ideas with each other, displayed in unit common areas.	Builds rapport, connection, and sense of common humanity
Clear Mutual Expectation	Patients and staff work together to create mutually agreed aspirations that apply to both groups equally.	Counters some power imbalances, creates a stronger sense of shared community
Mutual Help Meetings	Patients offer and receive mutual help and support through a daily, shared meeting.	Strengthens patient community, opportunity to give and receive help
Soft words	Staff take great care with their tone and use of collaborative language. Staff reduce the limits faced by patients, create flexible options, and use respect if limit setting is unavoidable.	Reduces a common flashpoint, builds respect, choice, and dignity
Positive Words	Staff say something positive in handover about each patient. Staff use psychological explanations to describe challenging actions.	Increases positive appreciation and helpful information for colleagues to work with patients
Bad News Mitigation	Staff understand, proactively plan for, and mitigate the effects of bad news received by patients.	Reduces impact of common flashpoints, offers extra support
Calm Down Methods	Staff support patients to draw on their strengths and use/learn coping skills before the use of PRN medication or containment.	Strengthen patient confidence and skills to cope with distress.
Talk Down	De-escalation process focuses on clarifying issues and finding solutions together. Staff maintain self-control, respect, and empathy.	Increases respect, collaboration and mutually positive outcomes
Reassurance	Staff touch base with every patient after every conflict on the unit and debrief as required.	Reduces a common flashpoint, increases patients’ sense of safety and security

Patient participation is an important part of the Safewards implementation, preferably in the form of a continuous co-creation process between staff and patients [22–24]. In cases where Safewards was successfully implemented, patients reported a stronger sense of community, safety and calm [5, 25]. They also described feeling less isolated, more hopeful and positive about their stay in addition to experiencing increased respect on the part of staff. In a study by Kennedy et al. [26], the implementation and possible improvement of the ten Safewards interventions were discussed from the perspective of consumers. It was concluded that although the model does not address important issues regarding the nature of involuntary treatment, the interventions may minimize harm and increase safety. Staff perceptions of Safewards have varied, from high and enthusiastic acceptance along with the belief that the model has a positive impact on conflict and containment, to poor participation and negative perceptions [21]. Staff working with patients who have intellectual disabilities reported positive experiences, such as fewer violent incidents and feeling safer [27]. They also described an increased sense of community with patients and were more positive about being part of the ward community. Several studies, including a randomized controlled trial, demonstrated a reduced incidence of violence and coercive measures after implementation of Safewards, while others did not show any significant effects [28]. In some studies, the extent of the reduction was attributed to the high fidelity of the implementation, which means that to a great degree the interventions were delivered by staff as intended [29–31].

Implementation fidelity is often measured to evaluate outcomes and better understand why an intervention is successful or unsuccessful [32]. It can be defined as to what degree an intervention or program is implemented as intended by those who developed it [32]. If an intervention lacks the expected outcomes, an evaluation of fidelity can indicate whether this is due to poor implementation or an inadequate intervention. In a complex intervention such as Safewards, the level of implementation fidelity can be influenced by many factors. Therefore, it is often recommended that those involved in implementation research and clinical development should collaborate with staff and patients within the healthcare system [33]. In a conceptual framework, Carroll [34] suggests that when evaluating implementation fidelity the focus should be on adherence. Adherence refers to the implementation adherence to the content, coverage, dose and duration of the intervention. Four potential modifiers will have an impact on the level of adherence: intervention complexity, facilitation strategies, quality of delivery, and participant responsiveness and capacity. The four adherence modifiers influence each other and there is evidence that for example quality of delivery is associated with participant responsiveness [35]. Participant responsiveness includes both those delivering and receiving the intervention and, in a health care context, refers to the willingness and ability of staff and patients to be involved and engaged. High patient responsiveness is achieved when patients are positive about and actively involved in the intervention. Furthermore, it entails patients’ positive perceptions of the acceptability, relevance, usefulness and outcomes of the intervention [32]. Given that many Safewards interventions require active patient participation, patient responsiveness is a crucial modifier for adherence and, consequently, the evaluation of implementation fidelity. For example, for Safewards to be implemented as intended and reach expected positive outcomes, the intervention ‘Discharge messages’ requires patients to write messages to other patients, ‘Mutual help meetings’ requires patients to actively participate and thank other patients, and ‘Know each other’ requires patients to write something about their personal hobbies and interests. At the same time, patient responsiveness and the quality of staff delivery of the interventions are mutually reinforcing adherence modifiers, in which high quality delivery by staff enhances patient responsiveness, and engaged patients contribute to better delivery quality by staff [36, 37].

The implementation fidelity of Safewards is often assessed by using the Safewards Fidelity Checklist (SFC), an instrument that mainly examines the number of interventions implemented by staff. In addition, there is an open text box in the SFC used for documenting the most significant staff responses to Safewards [38]. However, concerns have been raised about the SFC’s focus on objective and visible implementation evidence [29, 39, 40]. Moreover, the SFC does not include aspects of patient responsiveness, or patients’ perceptions of how Safewards interventions are implemented by staff [13, 32]. This may reflect a general lack of the patient perspective in the research on Safewards [21]. In one study however, the open text box in the SFC was modified to collect responses from both staff and patients [25]. Fidelity evaluation development is vital for the Safewards evidence base, as high-quality fidelity assessments affect study validity and can provide a deeper understanding of why Safewards is effective or not [40]. In this development, it is necessary to include patient responsiveness as an important fidelity modifier. The aim of the present study was therefore to investigate the Safewards implementation fidelity on an acute psychiatric ward from the perspective of patient responsiveness.

## Methods

### Setting and sample

The study was conducted in a 13-bed acute psychiatric inpatient ward, mainly for patients with affective disorders. The ward was chosen because the ward manager and team there had reported the successful implementation of the Safewards intervention. Coercive measures decreased by 75% and short-term sick leave among staff by 30%. Common diagnoses/syndromes were mood disorders, anxiety disorders, crises, personality disorders and neuropsychiatric conditions. The duration of care episodes averaged 11 days. The professional categories at the ward included specialized psychiatric nurses, registered nurses, assistant nurses, a psychiatrist, an assistant physician and a social worker. On the ward, patients received acute psychiatric care including psychiatric nursing, medical treatment, one-to-one support, psychoeducation and basic Dialectical Behavioral Therapy. Patients could also participate in activities such as walks, games and art. The psychiatric care at the ward focused on empowering people to take responsibility for their own abilities to deal with difficulties. Safewards supported the nursing staff in these efforts. The care was also moving towards a more person-centred approach during the implementation of Safewards.The ward manager was highly committed to the implementation of Safewards, and the team saw themselves as stable with positive group dynamics. At the time of our data collection they had implemented eight of the ten Safewards interventions over an almost three-year period in a co-creation process where they were divided into five groups. Each group was responsible for the implementation of two interventions.

Participants for the interviews were recruited by KC and GB, registered nurses at the ward at the time of the interviews and master students of psychiatric nursing, to become specialized psychiatric nurses. They wrote a master’s thesis in which they inductively analysed the interviews from a nursing perspective. The inclusion criteria were that the patient could speak Swedish, was able to provide informed consent to participate and should have been in the ward for at least five days in order to have experience of the care and interventions. First, general information about the study was presented by KC and GB to patients at a Mutual help meeting. No patient signed up for an interview after the information. The interviewers then recruited patients face-to-face at the ward after consultation with the ward manager to assess that the patients were capable to give their informed consent. Patients were provided with both verbal and written information regarding the study. This included details about the voluntary nature of participation, the purpose of the study, and the intended use of the data. Specifically, it was explained that the data would be utilized by students (the interviewers) for their master’s theses as well as by researchers for publications. The face-to-face recruitment resulted in ten people agreeing to participate, while three declined. No questions were posed about the reason for declining. We interviewed ten patients, one man and nine women, of whom four were aged 30 years or younger, four were between 31 and 40 and two 61–70 years.

### Data collection

KC and GB collected the data. As a first step, a modified version of the SFC (see Supplementary Material 1) [38] was used in order to assess the general implementation fidelity of Safewards on the ward. The SFC was filled in along with a detailed ward walkthrough observation of visible signs of Safewards, which were documented and commented on separately.

Subsequently, patient interviews were conducted over a 20-day period based on an interview guide that contained questions about seven of the ten interventions. The Positive words intervention was excluded due to the focus on the quality of staff handover content, which cannot be observed by patients.

The Soft words and Reassurance interventions are not reported in this article because they were not implemented at the time of the interviews. The patients were asked about their observations of manifest signs of Safewards as well as quality aspects of the interventions. Each intervention was briefly explained, and the participants were asked: (1) what they thought of the intervention, (2) about positive and negative experiences and (3) how the intervention could be improved (see Supplementary Material 2). The interviewers were instructed to use prompting, for example asking the patient to clarify what they meant by a statement, to obtain in-depth information. The interview guide functioned as intended at the first interview and no changes were made to it.

Seven of the patients were interviewed on the ward during their stay, and three who had been discharged agreed to be interviewed in a separate room next to the ward. The interviews, which lasted 26–85 min, were audio recorded and transcribed verbatim. They were performed in a single session, seven interviews were done by two interviewers and three interviews with only one interviewer present. Field notes were not taken as it was anticipated that the interviewers, who were actively working in the environment, would find it challenging to document these observations. The transcripts were not returned to the participants for comment. After ten interviews, patients gave similar information about how they perceived Safewards and enough of various kinds of events where Safewards had played a role.

### Analysis and interpretation

The Safewards interventions were used as categories. Within these categories, a qualitative descriptive text was written about the SFC and the walkthrough, while a qualitative descriptive analysis of the interview content was conducted [41–43]. We used the qualitative descriptive analysis method, as we aimed to obtain a straightforward qualitative description of patients’ responsiveness to the Safewards interventions [42]. The analysis started with GB and VP reading the transcripts to gain an overview of the content of the interviews. GB summarized every patient’s view of the seven interventions based on the interview guide. VP merged these summaries into a single summary, which was discussed with AB and LK. VP read through all the interviews to add more relevant information and suitable quotations to the result section. All co-authors commented on the results section and which quotations were the most relevant. The names in the quotations were changed and the pronoun ”she” was used for all participants to protect their identity. The participants were not asked to provide feedback on the findings.

## Results

In general, the findings indicated that the patients had noted the implementation of Safewards and were positive about it. Some gave examples of improvements that had happened over time or of the ward being better than other wards. They expressed that staff now had a more positive attitude when interacting with patients. The ward and staff were perceived as welcoming, familiar, supportive and felt safer. The patients felt respected, less alone and more hopeful. All these perceptions seemed to contribute to the patients’ experiences of the ward as a safe environment. They also expressed taking responsibility for other patients and the ward climate in general.

In this section, we first provide a brief description of the Safewards implementation fidelity as revealed by the SFC and the walkthrough. Thereafter we present the patients’ responsiveness to Safewards. A summary of the findings is presented in Table 2.

**Table 2:** Overview of fidelity findings from the SFC, ward walk-through and patient interviews

Intervention	Observation SFC^1^/walkthrough	Patient responsiveness	Patients’ s comments on aspects that require improvement
Discharge Messages	Discharge tree on corridor wall. 27 messages. Brochure.	Thoughts about own message to fellow patients. New insights. Hope and encouragement.	Noisy environment around the tree. Too much gratitude to staff.
Know Each Other	Folders presenting the 23 staff members. Whiteboards outside patient rooms, no patient presentations.	Opportunity for patients to express how they want to be treated by others. Facilitating daily conversations. Feeling respected. Less distance and power difference between patients and staff. Fear of being exposed, risk of prejudice.	Lack of information about the intervention. Information sometimes not up to date. Less pre-defined suggested categories on the sheet to be filled in.
Clear Mutual Expectation	Several posters illustrating the intervention on the ward.	Promoting respect and responsibility. Creating a safer ward environment. Less power difference between patients and staff. Some patients too ill to meet the expectations stated on the poster.	Staff could be better at living up to expectations. Poster could be easier to read and include pictures. More information about the intervention at Mutual help meetings.
Mutual Help Meetings	Meetings every weekday morning. Folder with meeting structure. Visible patient information about meetings.	The meetings were perceived as informative, helpful and motivating. It took time getting used to actively participating in these meetings.	The structure of the meetings varied too much, depending on the chairperson. The meeting should mainly focus on positive things.
Bad News Mitigation	Intervention headline in digital handover agenda. Information on table in conference room. Box of treats prepared for bad news meetings with patients.	Staff noticed when patients had received bad news and supported them. It made patients feel safe, be more communicative and less lonely.	Being unable to see your relatives because of Covid 19 felt like bad news that did not have a proper explanation.
Calm Down Methods	Sensory room and calm down items available in cabinet Visible “emotion map” on a wall	Feeling of safety. Sensory room, calm down items and ward activities helpful. Staff noticed if patients needed help. Staff helped patients to develop strategies to handle emotions. Felt respected and listened to. Reluctance to bother staff.	The Covid 19 pandemic meant that hugs were not allowed, which was difficult. Patients could feel that emotions must be hidden from staff. A wish for faster support, despite busy staff.
Talk Down	Poster visible for staff Regular training sessions	Staff handled incidents calmly and efficiently, not escalating the situation.	Despite staff competence it could feel unsafe to witness aggressive situations.

﻿1). The Safewards Fidelity Checklist (SFC)

### Discharge messages

#### SFC/walkthrough

There was a big tree painted on the wall with discharge messages in the corridor opposite the ward entrance. Patients were asked to leave a message in connection with their discharge. At the time of the fidelity check, there were 27 discharge messages and a brochure with information about the intervention.

#### Patient responsiveness

It was great, it’s the first thing you [as a patient] see, there were so many beautiful leaves, just being able to read it when you’re standing outside the nurses’ station waiting …. That was also something that I noticed right away when I walked in, literally the first thing… Pat. 7.

Most patients considered the location of the discharge tree to be appropriate, that it was aesthetically beautiful, and that they took the time to read the messages. A patient described how she “naturally” drawn towards the tree because of its location and often stood there waiting for medicine or staff. The tree gave comfort during bad days, as it was hopeful and invigorating to read that others had received help from staff and recovered, which created a sense of safety. It also sparked a curiosity about previous patients: What happened after discharge? Some gained a new perspective on how to approach their problems, while others just learned to accept the situation, trusting that it will improve and letting their recovery take time. It was important for patients that the messages were positive and encouraging. Sometimes they could think long and hard about what they wanted to convey when it was time for discharge.

Some felt that the environment around the discharge tree was often too noisy, a lot of people passing by all the time, which made it difficult to absorb the messages. One patient thought that her fellow patients should thank themselves and not the staff.

### Know each other

#### SFC/walkthrough

There were two folders in the common area of the ward presenting each of the 23 staff members on a separate page. Patients had a small whiteboard with pre-defined suggested categories outside their rooms where they could write about themselves. However, there were no written know-each-other messages from patients at the time of the walkthrough. The reason for using a board instead of pages in a folder was that for most patients the care episodes were relatively short.

#### Patient responsiveness

It gave more hope in a way …. That you [staff members] really show who you are and that you are passionate about your work. Yes, I really felt like I had come to the right ward when I read your folder [with presentations]. Pat. 3.

It felt welcoming that staff had made an effort to create the folder with information about their interests and other personal details. Knowing something about the staff members who were working at the ward contributed to safety. It also facilitated daily communication and made it easier to ask for help. The fact that the presentations were always available meant that patients could learn about staff members and fellow patients at their own pace. Some had read the staff presentations several times. The intervention reduced the power difference between staff and patients because staff members became persons and not “just their nursing scrubs”. A patient reported feeling touched by the fact that staff presented themselves. It felt familiar and positive.

At the time of the interviews, some patients had written about themselves on the whiteboard outside their room. They thought that it was especially fun and interesting to read about fellow patients. A patient who presented herself on the whiteboard felt respected and appreciated reading others’ presentations. Even those who wanted “to be a little anonymous” or did not consider it necessary to write about themselves appreciated the possibility. They thought it was good that patients could write about how they, for example, wanted to be treated by others. Some patients considered that it was difficult to expose oneself in a presentation when feeling unwell and could be reluctant to present themselves “fully” because of the risk of prejudice.

At times it felt a little less safe to be able to do it, there can be a lot of prejudice about me because …. I do this and I do that. I sometimes felt a little …. tingle in my stomach maybe, but then I thought – No, to hell with it, I can be myself and it may seem very crazy and all. I care less about that because I’m here to help as well, to improve this system. Pat. 6.

There were several similar statements where patients expressed that they wanted to take responsibility for the ward climate and safety. Patients had ideas about how to improve the intervention, for example by providing more information about the intervention and its purpose, that it had too many pre-defined suggested categories to choose from and that the folders should be updated with new information about and pictures of those staff members who were not yet included in the presentations. A patient thought that the staff only revealed “their good side” and should also inform about their weaknesses.

### Clear mutual expectations

#### SFC/walkthrough

There were several posters in the ward pertaining to Clear mutual expectations. Before the implementation started, interviews were conducted by a Peer support person focusing on this topic with patients.

#### Patient responsiveness

Patients had seen the poster and been informed about the intervention. They expressed that the expectations promoted mutual respect and taking responsibility for the ward environment without aggressive behaviour. Several patients mentioned that they could go to the poster when they lacked information and could also help their fellow patients to adhere to rules so as not to annoy staff. Some were of the opinion that everyone must take responsibility and that some people may need to be reminded of it. They thought that the mutual expectations contributed to a good and safe environment and appreciated that the staff had made an effort to create the expectations, which gave them hope.

I have both good and bad experiences of inpatient care and care in general, but it was hopeful because it has been difficult to be cared for as an inpatient, but this was like physical evidence that you [staff members] actively work to make it better, somehow. And it gave me hope …., it made me a bit calmer that “Okay, maybe this time it can be different” so …. to make changes in health care is quite (laughter) difficult and big, so just managing to get the posters printed and put them up means a hell of work. So .... that was helpful.

Patients also pointed out that it can be difficult to have clear mutual expectations in an environment where people are so sick, but that it is a good strategy to encourage everyone to take responsibility. The intervention reduced the power difference between staff members and patients.

Patients made some suggestions for improvement, for example keeping promises, as staff members sometimes made promises that were either fulfilled late or not at all. Other suggestions were using a different font to make the poster easier to read and making patients aware of the intervention by providing more information about it at the Mutual help meetings. 

You [staff] could show it differently, not just text. I don’t know what it could be but something, pictures, photographs …. Yes, a picture and text I think, because a picture can be associated with [something] and I will be more likely to remember it. Pat. 10.

### Mutual help meetings

#### SFC/walkthrough

The staff arranged Mutual help meetings every weekday morning after breakfast in the dining room. There was a folder for staff use in which the structure of the meetings was described. Patient information was also displayed prominently in the ward.

#### Patient responsiveness

A number of patients found the morning meetings beneficial and helpful, as they provided an overview of the day. At the meeting, everyone was given the opportunity to talk about issues that felt difficult but also to express positive feelings. It was a good forum for asking about something or gaining information about what would happen during the day. The opportunity to express gratitude at the meeting was considered a good start to the day.

…and it was great that the staff brought up, for example, “It was really hard to get to work because it was raining …. but I’m grateful that I’m here now”. And it felt great that they are grateful that they are here …. And I thought that was among the best things I experienced in the ward. Pat. 6.

The fact that the staff dared to bring up subjects that they thought were problematic and what they were grateful for made it easier for patients to become more communicative. It was difficult for patients to know what was appropriate and how much to open up and communicate about their personal life. Some of them stated that they wished to remain anonymous during their stay in the ward. Talking to other patients made it easier to be open, which could be especially difficult on days when they felt very unwell. They reported that they sat relatively quietly and just listened at the first few meetings, but later became more communicative. Patients thought that the meetings motivated them and made them feel less alone. It felt positive that everyone wished each other a good day. These meetings helped to create a social community in the ward.

Yes, often it is …. you [the chair, a staff member] present which day it is and the date and who has a name day …. And then there is a round where everyone gets to express what they think, that they wish everyone a good day, that they are grateful to be here …. or you can say that you would like help to withdraw money. And then it’s written down and …. it feels good that everyone wishes each other a good day, I think, it’s a nice little start to the day. Pat. 9.

Having the meeting early in the morning was experienced as both positive and negative. It was nice to sit and have breakfast in peace and quiet, hence attending the meeting was an effort but at the same time it was positive to obtain information about the day. As a patient, there was a risk of feeling pressurized into participating when the meeting started immediately after breakfast in the common dining room. There were patients who often overslept and therefore did not attend many meetings.

Patients had suggestions for improvements. They noted that each chairperson structured the meeting differently, which was frustrating. They wanted the structure of the meetings to remain more or less the same. Hearing about other people’s problems could be negative for their own well-being and therefore they stated that the meetings should mainly focus on positive aspects.

### Bad news mitigation

#### SFC/walkthrough

One of the headings in the digital agenda for handover reports was Bad news mitigation aimed at routinely raising the question of whether any patients had received or might receive bad news. A laminated information sheet about how patients who had received bad news should be dealt with was on the table in the conference room where the handovers took place. There was also a box of “treats”, such as tea and biscuits, which could be used during Bad news mitigation meetings with patients.

#### Patient responsiveness

Some patients reported receiving bad news that was difficult to handle, but that they had received good support from staff.

Well, when I was hospitalized last time, my grandfather was very, very ill and …. Then I got support from the staff when, when I was informed about that …. Yes, we sat and talked and so …. Yes, it was good. Pat. 10.

There were also patients who did not receive any bad news themselves but who observed others having done so. They stated that their fellow patients had received compassionate and empathetic care. Patients experienced that staff noticed when they needed to talk about something or required support in a tricky situation. Staff support made them feel safe, more communicative and less lonely.

I experienced that you [staff members] are present when, for instance, you speak in a way that the person really understands and that you are really there, also some minutes afterwards, so that the person calms down and really understands what has happened and you speak in a calm voice. Pat. 2.

According to the patients, an example of bad news was that relatives were not welcome at the ward during the Covid-19 pandemic. It was difficult for staff to explain why friends and family were not allowed to visit patients. In such a case, it would have been helpful to receive accurate information about the situation; otherwise, patients might think that it was their own fault and would develop “dark thoughts”.

### Calm down methods

#### SFC/walkthrough

The ward had a sensory room and a calm down cabinet, with many different sensory items and equipment that could be used for calming purposes, like hug chair, weight vest and blanket, ice pack and a Star Projector. In addition, there was an “emotion-map” on the wall containing a description of emotions and their functions as well as suggestions for calming strategies that could be used to cope with various emotions.

#### Patient responsiveness

I was stressed and then I was given a heated cushion, because it could help me handle anxiety, and it did, I never thought of that myself before. Pat. 5.

The calm down methods were appreciated by patients, many of whom perceived that the intervention worked well and created a feeling of safety. Patients expressed that they had been helped by the sensory room, a quiet place to calm down in, or by items from the calm down cabinet. They also reported that staff helped them to find strategies to deal with anxiety and emotions. Some patients also noticed that staff members helped fellow patients to calm down. They found it positive that staff noticed patients who were having a hard time and tried to find ways to help them calm down.

Patients considered the activities organized in the ward, including playing games, going for walks together and music quizzes, as very helpful ways to handle difficult emotions. The activities made patients feel seen, heard, and less alone in difficult situations. Patients described working on their crisis plan together with staff. Even before admission some had several strategies for independently managing to calm challenging emotions. They felt that staff respected them, listened, reminded them about their strategies and provided support for finding new strategies. In some situations, patients could find receiving help difficult and frustrating, although it was often perceived as valuable. Below is a quotation from a patient who received help from staff to write a list of strategies she could use when needing to calm down.

I thought it was very good that when I had a very severe anxiety attack, someone asked “Okay, but where are you on your list?” …. I had a copy by my bed so I could check it …. and it was very nice that, for instance, I was allowed to go out and smoke even though it wasn’t smoking time because a cigarette calms me down, like, that someone took the time to do it. I think that I was listened to and the staff reminded me of skills that I know work for me. Pat. 7.

One patient did not seem to know about the intervention. She said that patients must not show emotions in psychiatry because of the risk of being medicated, instead of being offered a chat or a hug. The need for social distancing during the pandemic was difficult for those who liked getting a hug. This patient used her own strategies without talking to staff. Another patient had sometimes wished to receive support more quickly but said that she understood that staff had a lot to do.

### Talk Down

#### SFC/walkthrough

A Talk Down poster was visible to staff. Every two weeks staff members practised the talk down intervention in training sessions with role play.

#### Patient responsiveness

So, I noticed that staff knew exactly what to do when it happened, … trying to punch or break free from a hold, when everyone joins up and helps, talks calmly and methodically to the person and then someone tells everyone else to go to their rooms while you [staff member] help to calm that person down. Pat. 2.

While the participants did not report being involved in a de-escalation process, they described how staff members managed aggressive behaviour from patients as well as an accident. Staff members did so calmly and efficiently to ensure that the situation would not escalate. Patients also observed that staff sometimes restricted patients in order to prevent them from creating trouble for other patients.

So, there are many situations all the time. It’s good that you [staff] try to be as flexible as possible so that it doesn’t create chain reactions …. That we kind of feel worse as a result. For instance, if someone has to be put in restraints, that it is not …. It may not be very dignified and therefore better that others don’t see it. Pat. 10.

The patients’ care episodes differed, for some it was chaotic, and they had seen events that were difficult or unpleasant, which created a feeling of lack of safety. Even if they themselves could handle their emotions, they found it difficult to witness when others felt unwell. Some patients described that the ward was calm during their own care episode, while others only experienced a single situation where a fellow patient was aggressive.

## Discussion

To summarize, the findings indicate high implementation fidelity, both in terms of the SFC and the patients’ responsiveness to Safewards. The seven interventions implemented were all clearly observable at the SFC/walkthrough. Evaluation of patient responsiveness to the implementation of Safewards may differ from that of patients’ experiences of Safewards in general. This is because responsiveness more specifically refers to patients’ enthusiasm and engagement, in addition to their perception of the acceptability, usefulness and relevance of Safewards. The results of the present study contain many examples of how patients describe responsiveness based on these attributes. For example, patients expressed that the discharge tree provided comfort during bad days, as it created hope (usefulness). The attached Know each other folder could also make them familiar with staff members at their own pace (acceptability) and one patient heard a staff member at a Mutual help meeting saying that she was grateful to be on the ward, which was described as one of the best things that particular patient had experienced on the ward (enthusiasm).

The patients described staff behaviour and the ward climate as positive, and that the interventions and other activities involving staff members created a feeling of safety and could distract from difficult thoughts and feelings. They felt respected, less alone, hopeful and safe. This is similar to the results of Maguire et al. [25] and Fletcher et al. [5], who described patients’ feelings of hope, safety, respectful relationships and sense of community on wards in which Safewards was implemented. In our study, the patients more clearly emphasized that they took responsibility for others and the ward environment than was the case in the aforementioned studies. Some patients were surprised that they were expected to take responsibility, while others considered it a matter of course. Although not fully clear, it is possible that the interventions involving Clear mutual expectations and Mutual help meetings could have encouraged patients to take a more active role in ward responsibilities and supporting others, or to realize that such behaviours were appreciated on the ward. There is a lack of research on assuming responsibility and naturally occurring peer support among patients [44]. However, our study indicates that patient engagement in Safewards may enhance opportunities for self-help and peer support, both within Safewards interventions (e.g., Calm Down methods and Mutual Help Meetings) and through spontaneous initiatives. In this way, active patient participation in the implementation of Safewards appears to encourage empowerment and support recovery processes which in turn may reduce conflict and containment [45].

Several participants experienced it as challenging being in a ward where so much communication between patients and staff as well as among the patients themselves was expected, for example through Mutual help meetings and Discharge messages. Patient engagement was demonstrated by several communication related improvement suggestions made about Safewards, such as more accessible information by means of simplified text or use of pictures. Similarly, an important factor in implementation is dosage, meaning patients’ level of exposure to the interventions [46]. A participant in this study had previously experienced that it was necessary to adapt to the ward rules and routines and not show negative emotions to avoid the risk of coercive measures. As this patient did not exhibit anxiety to staff members, no Calm down methods were used. Several studies indicate that many patients have similar thoughts [6, 47–50]. Therefore, it is important to ensure that the interventions are sensitive to individual patient needs and that person centredness is not compromised.

The present study is a first attempt to examine implementation fidelity to Safewards interventions by focusing on patient responsiveness. Including participant responsiveness when measuring implementation fidelity is important especially in complex interventions. In a review of complex rehabilitation interventions that examined 43 studies from a theoretical implementation perspective, the responsiveness of the participants, both staff and patients, was the most frequently mentioned factor affecting fidelity [51]. In research on Safewards, the focus has often been on the general response of staff and sometimes patients, as opposed to their response to specific interventions. When focusing on the responsiveness to the different interventions, we also gained information about strategies to facilitate implementation, delivery quality and adherence [32]. It became clear that despite Safewards, staff occasionally seemed to have difficulties dealing with certain situations. Patients who observed this could perceive the ward as an unsafe environment.

According to the patients, inconsistencies in staff behaviour and different ways of implementing the interventions affected the quality of delivery; this was particularly obvious in the Mutual help meeting intervention. Hence, staff responsiveness to, and way of working with, the Safewards interventions had a direct impact on patients’ responsiveness to them and whether or not they perceived them as helpful. It has been suggested that participant responsiveness may have a major impact on fidelity, and the connection between staff and patient responsiveness has been described by Carroll et al. [32] as a key aspect of implementation. This connection may be of particular importance when implementing an intervention such as Safewards, which aims at reducing levels of conflict and containment. Providing a therapeutic ward environment, including therapeutic engagement by staff in collaboration with patients, have been described as central to the reduction of conflict and restrictive practices, as well as to the quality of mental health nursing practice [52, 53]. Therefore, for staff to implement Safewards in a task-orientated and instrumental fashion without positive responsiveness is unlikely to be successful.

An important determinant of successful implementation of an intervention is local and organizational leadership [54]. Findings from our study suggest that leaders responsible for the implementation of Safewards, need to recognize the importance of facilitating for a positive patient and staff responsiveness. For example, they should be aware of any negative conclusions about the effectiveness of Safewards based solely on observations of staff performing Safewards activities without identifying a lack of positive responsiveness. Therefore, it is likely that the implementation of Safewards would benefit from leaders actively supporting this aspect in their implementation strategies.

### Strengths and limitations

Our study adds to the sparse literature on patient experiences of the Safewards model. The results are relevant for clinical practice when implementing Safewards, as they provide insights into patient responsiveness to and suggestions about how to improve specific Safewards interventions. Possible pitfalls in the implementation process may thereby be avoided.

A main limitation is that the study was conducted at only one ward, mainly comprising patients treated for affective disorders. Interviews from other wards with different patient profiles may have contributed more knowledge about patient responsiveness and further suggestions for improvement. Another limitation is that observations, recruitment of participants and interviews were conducted by staff employed at the ward, possibly leading to bias in observations, selection of patients, and their responses to the interview questions. As the interviewers had been engaged in the implementation of Safewards at the ward, they may have reported more observations on the SFC than an independent researcher would have done and may also have selected favourably disposed patients for interview. Some patients were interviewed while still on the ward in a dependent situation. We handled this bias by giving clear instructions to the interviewers and the ward manager about which patients could be interviewed, how to use the SFC and the interview guide. Clear questions in the interview guide were posed to encourage patients to be critical and make suggestions for improvement. These were single session interviews. We did not contact the patients again, even though it could have provided valuable knowledge. We saw it as both ethically and practically problematic. Reminding people of their time on a psychiatric ward can arouse many emotions and it is often difficult to get in touch with former patients. On the other hand, it is a strength that the interviewers were very familiar with the work at the ward and had wide experience of communicating with patients, possibly leading to richer responses. After the information at the Help meeting no patient immediately signed up for an interview. Some patients may have a lack of energy, and some may not dare to speak or ask questions in front of others in a group. However, the interviewers noticed that all participating patients wanted to take part of the study and express their thoughts about their stay at the ward. Despite the shift in direction towards becoming a more patient-centred ward, many suggestions for improvement emerged that the staff had not previously received from patients. To increase trustworthiness all authors took part in the analysis. The authors possess diverse backgrounds, including mental health nursing, social work, and psychiatric health services research. The primary author also has personal experience with psychiatric inpatient care as a patient. These varied competencies and experiences enhance the potential for multiple perspectives in analysing the interview material.

## Conclusions

This study confirms previous research that patient responsiveness is an important factor in the assessment of fidelity in prevention programs, such as Safewards. The patients’ descriptions of the acceptability, relevance and usefulness of the specific interventions, to a high degree reflected the objective visual observations made through the SFC and ward walkthrough. Patient engagement was demonstrated by several suggestions about how to adapt the interventions in order to make them more useful and accessible. This shows a potential to obtain valuable input from patients when implementing and adapting Safewards in a ward and to achieve high quality implementation and levels of fidelity. This study is also clinically relevant as it presents many examples of practical work with these interventions and their effects on patients’ experiences of care.

### Electronic supplementary material

Below is the link to the electronic supplementary material.

Supplementary Material 1

Supplementary Material 2

## Data Availability

The data are not available because it could compromise the individual privacy of participants. They are stored at the University Health Care Research Center, Region Örebro County, and may be requested by other researchers.

## Abbreviations

UN: United Nations
EU: European Union
PRN: Pro Re Nata. A medication prescribed to treat short term or intermittent medical conditions, not to be taken regularly
SFC: The Safewards Fidelity Checklist
WHO: World Health Organization
