# Supplementary material for: Patient responsiveness as a safewards fidelity indicator: a qualitative interview study on an acute psychiatric in-patient ward
Source: BMC Health Serv Res. 2024 Aug 12;24:922. doi: 10.1186/s12913-024-11326-z (PMC11321007; doi:10.1186/s12913-024-11326-z)
Supplement: Supplementary file 1 — Supplementary Material 1 [file 12913_2024_11326_MOESM1_ESM.docx]

Additional file 1


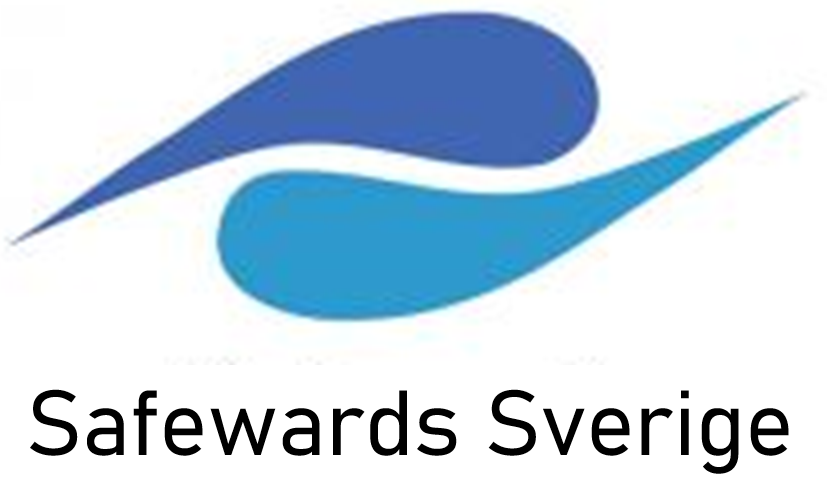


**Safewards**

**Checklist for fidelity**

| Date: | Ward: | Observer: |
| --- | --- | --- |

*Start by marking the interventions that have been implemented according to information from the ward. Only these should be assessed.*

| **Intervention** | **Minimum visible observation** | **Yes** | **No** |
| --- | --- | --- | --- |
| Clear Mutual Expectations | There is a “Mutual Expectations” poster on the wall publicly.  *Comment:* |  |  |
| Know Each Other | A "Know Each Other" binder/folder or another form of presentation is available in a public area of the ward.  *Comment:* |  |  |
| Mutual Help Meetings | Some form of documentation supports that during the last month, meetings were held at least once a week.  *Comment:* |  |  |
| Discharge Messages | There is a noticeboard or something else for Discharge Messages visible to patients and there are messages from patients there.  *Comment:* |  |  |
| Positive Words | A visible Positive Words poster is located in at least one room used for reporting.  *Comment:* |  |  |
| Soft words | A Soft words poster is displayed in the staff areas.  *Comment:* |  |  |
| Bad news mitigation | Some form of documentation on how the intervention is to be carried out is readily available for staff.  *Comment:* |  |  |
| Calm Down methods | There is a sign visible to patients informing them of the intervention as well as a range of calming items for patients to use.  *Comment:* |  |  |
| Talk down | At least one Talk Down poster is displayed on a wall visible to ward staff.  *Comment:* |  |  |
| Reassurance | Some form of documentation on how the intervention is to be carried out is readily available for staff.  *Comment:* |  |  |
