# Supplementary material for: Patient responsiveness as a safewards fidelity indicator: a qualitative interview study on an acute psychiatric in-patient ward
Source: BMC Health Serv Res. 2024 Aug 12;24:922. doi: 10.1186/s12913-024-11326-z (PMC11321007; doi:10.1186/s12913-024-11326-z)
Supplement: Supplementary file 2 — Supplementary Material 2 [file 12913_2024_11326_MOESM2_ESM.docx]

Interview guide

*Clear Mutual Expectations*. There is a poster where we inform about our mutual expectations for the ward.

- What do you know about these mutual expectations?
- How have the mutual expectations affected you?
- What could we do to improve this intervention?

*Know Each Other.* There is a folder with a presentation of staff and a Whiteboard for the patient to introduce her-/himself.

- What do you know about the intervention Know Each Other and how has it affected you?
- How do you perceive that staff present themselves?
- How do you perceive to present yourself?
- What could we do to improve the intervention?

*Discharge Messages.* There is a tree with Discharge messages with the purpose to create hope.

- What is your experience of the intervention?
- How has reading other people's Discharge Messages affected you?
- What can we do to improve the tree and the intervention?

*Mutual Help Meeting.* We often have a Mutual Help Meeting to inform each other about the situation and to support each other.

- What is your experience of participating in Mutual Help Meetings?
- How did you feel about attending these meetings?
- What could we do to improve the meetings?

*Soft Words.* The staff should have a gentle manner when asking patients to do something or to stop doing something that might disturb others.

- What are your experiences of staff asking you or others to do or stop doing something?
- How did you feel about it?
- How did you experience the course of events?
- What can we do to improve the intervention Soft Words?

*Bad News Mitigation.* If a patient receives negative news, the staff on the ward should provide support in a reassuring manner.

- What is your experience of Bad News Mitigation?
- How was the course of events?
- How do you feel that the staff supported you or someone else?
- What can we do to improve the intervention Bad News Mitigation?

*Calm Down Methods.* If a patient has difficulties with her/his emotions, the staff should help the patient with Calm Down Methods, e.g. a distraction intervention.

- What are your experiences of staff helping you with calming down methods?
- How did the staff have respond to your or another patient's needs?
- How was the course of events?
- How can we improve the intervention Calm Down Methods?

*Talk Down.* The staff should deal with upset, anxious or aggressive patients in a calm and understanding manner in order to de-escalate the situation.

- What are your experiences of staff de.escalating difficult situations?
- How did you feel about the course of events?
- How did it affect you?
- How do the staff treat you or another patient in the situation?
- What could we do more to improve the intervention Talk Down?

*Reassurance.* If something has happened in the ward that can be perceived as frightening by patients, for example when a patient feels particularly unwell and things have become uneasy in the ward, the staff must talk to all patients about it and inform them of the situation. The aim is for everyone to feel safe.

- What are your experiences of being scared or anxious on the ward?
- How was the course of events?
- How did the staff act?
- How did the staff respond to your or others' needs for support?
- How can we improve the intervention of Reassurance?

*One last question.* If you see all these interventions that we talked about during the interview:

- How have they affected your sense of safety in the ward?
- What can the staff do more to increase the feeling of security in the ward?
